# Intraoral Immature Malignant Teratoma with No Evidence of Other Sites of Involvement in a 6-Year-Old Patient: A Case Report

**DOI:** 10.3390/reports8010003

**Published:** 2024-12-27

**Authors:** Vasileios Zisis, Christina Charisi, Konstantinos Poulopoulos, Petros Papadopoulos, Athanasios Poulopoulos

**Affiliations:** Department of Oral Medicine/Pathology 1, School of Dentistry, Aristotle University of Thessaloniki, 54124 Thessaloniki, Greeceakpoul@dent.auth.gr (A.P.)

**Keywords:** teratoma, malignant, immature, oral, intraoral, cancer, tumor, child, juvenile, head and neck

## Abstract

**Background and Clinical Significance**: Head and neck teratomas are embryonal tumors that develop when totipotent germ cells escape the developmental control of primary organizers and form a more-or-less organoid mass in which tissues from all three germ layers (ectoderm, endoderm, and mesoderm) can be identified. Mature teratomas may either transit into germ cell or non-germ cell malignancies or remain histologically mature with the possibility of growing, thus inducing certain complications when reaching a large size. This article aims to investigate a very rare case of a 6-year-old child who exhibited a recurrent intraoral mass with multiple conflicting biopsies. **Case Presentation**: A 6-year-old male patient was referred to the postgraduate clinic of the Department of Oral Medicine/Pathology, Dental School, Faculty of Health Sciences, Aristotle University of Thessaloniki, Greece, because his pediatric dentist noticed an exophytic, intraoral mass, distal to tooth #75 during a routine checkup. The first histopathological examination showed a gingival tumor, classified as a small round blue cell tumor, with greater similarity to adamantinoma-like Ewing sarcoma (ALES) and less to synovial sarcoma. The second pathologist examined the same tissue specimen and suggested the extremely rare presence of an immature malignant teratoma. Following chemotherapy, the rest of the teratoma with the adjacent tooth #75 was removed, and the histopathological examination showed a mature teratoma. **Conclusions**: This case illustrates the crucial role of the dentist, and in this case of the pediatric dentist, to promptly diagnose the underlying disease. Genetic screening may assist in detecting high-risk populations. In such complex histopathological cases, the importance of cooperating with experienced oral and maxillofacial pathologists is highlighted. We describe a rare case of intraoral malignant teratoma, and an extended literature review revealed that our case is the first ever reported.

## 1. Introduction and Clinical Significance

Head and neck teratomas are embryonal tumors that develop when totipotent germ cells escape the developmental control of primary organizers and form a more-or-less organoid mass in which tissues from all three germ layers (ectoderm, endoderm, and mesoderm) can be identified [1]. Their histological features are therefore heterogeneous and can include cystic or solid areas with an organoid pattern and mature or immature components. Teratomas of the head and neck region account for 6% of all teratomas [2]. In children, they are usually diagnosed early because of common symptoms such as shortness of breath, facial dysmorphism, and orbital involvement [1,3]. Unlike adults, teratomas in children are usually congenital and rarely become malignant. Mature teratomas may either transit into germ cell or non-germ cell malignancies or remain histologically mature with the possibility of growing, thus inducing certain complications when reaching a large size [4]. Complete surgical excision should be preferred, and if not possible, multiple biopsies from different sites should be carried out to ensure the full histopathological examination of the lesion and to avoid any regional malignant transformation. This article aims to investigate a very rare case of a 6-year-old child who exhibited a recurrent intraoral mass with multiple conflicting biopsies.

## 2. Case Presentation

**Figure 1:**
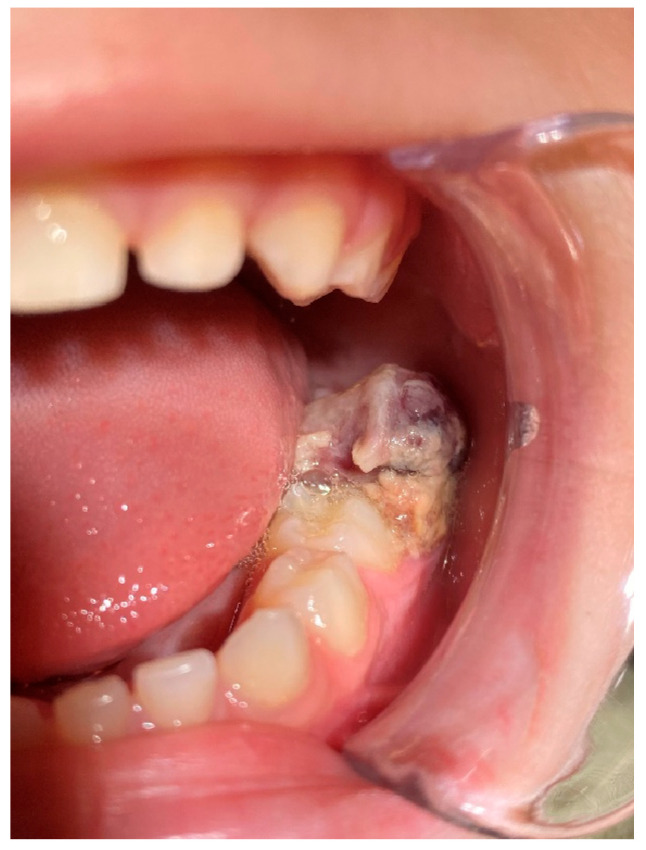
Intraoral depiction of the tumor.

A 6-year-old male patient was referred to the postgraduate clinic of the Department of Oral Medicine/Pathology, Dental School, Faculty of Health Sciences, Aristotle University of Thessaloniki, Greece, because his pediatric dentist noticed an exophytic intraoral mass during a routine checkup. His mother provided informed consent, and the examination followed. The medical history revealed no underlying conditions or medications, and the patient reported mild pain in the area under investigation for the past two weeks. Over the past few days, he was unable to close his mouth properly because the mass interfered with his normal occlusion. The clinical examination showed a soft, dark-colored tumor on the alveolar process, distal to tooth #75 (Figure 1).

A full excisional biopsy followed, and the tissue specimen, with a diameter of 1 cm, was subjected to histopathological examination.

**Figure 2:**
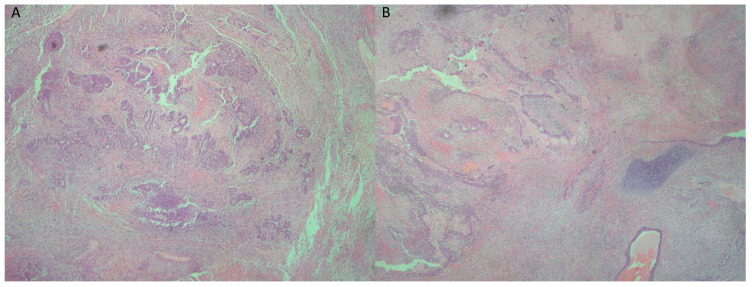
(**A**) Neoplastic adenoid and solid neuroepithelial formations within immature mesenchyme (hematoxylin–eosin ×40). (**B**) Neoplastic adenoid and solid neuroepithelial formations within immature mesenchyme, with accompanying presence of islands of mature cartilage (right) (hematoxylin–eosin ×40).

**Figure 3:**
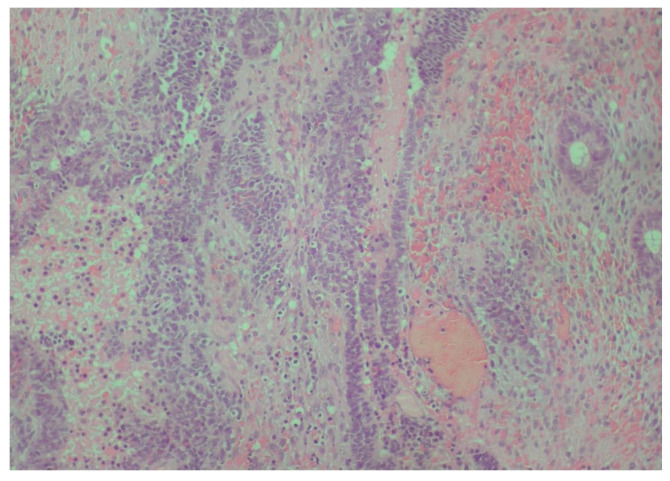
Neoplastic adenoid and solid neuroepithelial formations characterized by relatively uniform, hyperchromatic nuclei, with moderate atypia and several mitoses (hematoxylin–eosin ×100).

**Figure 4:**
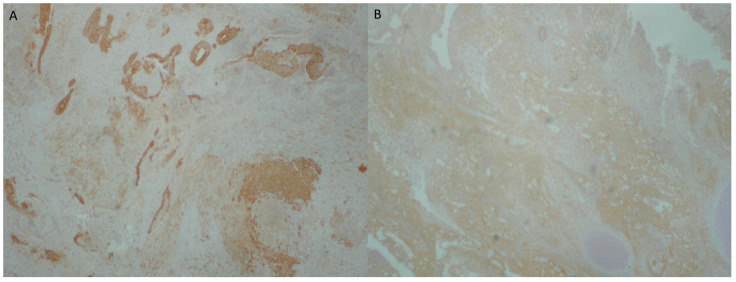
(**A**) Intense and diffuse positivity of the neoplastic adenoid formations in broad-spectrum cytokeratins AE1/AE3 (immunoperoxidase ×40). (**B**) Intense and diffuse positivity of neoplastic cells for CD99 (×40 immunoperoxidase).

**Figure 5:**
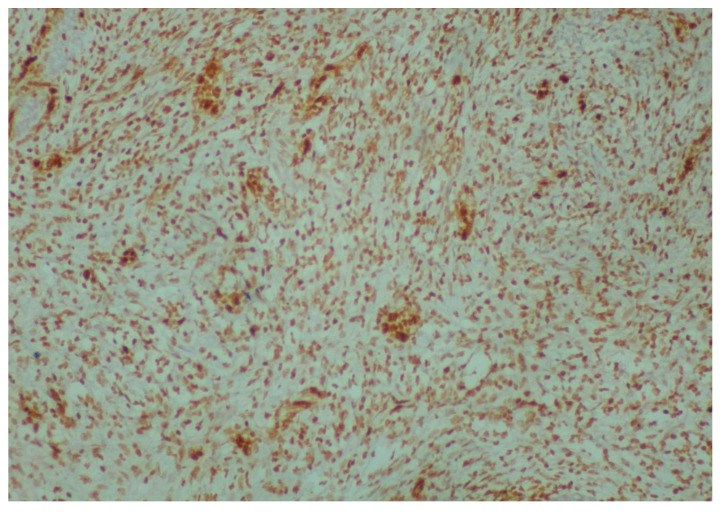
Intense and diffuse ERG (immunoperoxidase ×100) positivity of immature mesenchymal cells.

**Figure 6:**
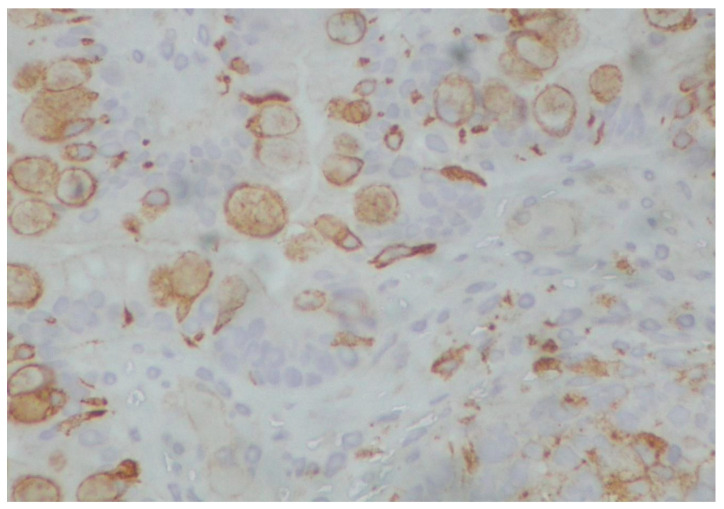
Synaptophysin (immunoperoxidase ×400) positivity of scattered neuroepithelial neoplastic cells.

The first histopathological examination showed a gingival tumor, classified as a small round blue cell tumor, with greater similarity to adamantinoma-like Ewing sarcoma (ALES) and less to synovial sarcoma (Figure 2, Figure 3, Figure 4, Figure 5 and Figure 6).

**Figure 7:**
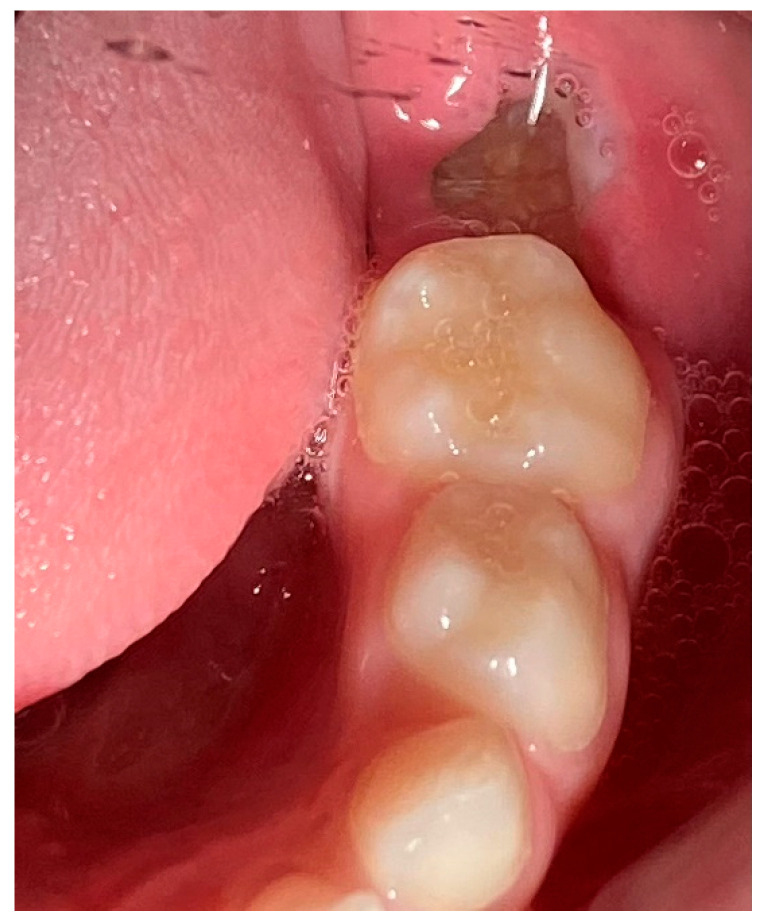
Postoperative clinical imaging after 7 days. The wound healing progresses uneventfully.

The patient returned for the follow-up after 7 days, and the wound healing had proceeded uneventfully (Figure 7).

**Figure 8:**
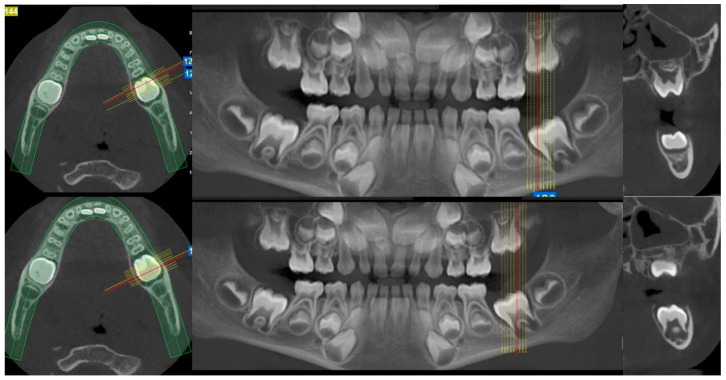
The CBCT examination did not reveal any bone involvement.

Subsequently, the patient was referred for a cone beam computed tomography (CBCT) examination to establish whether the mandible was involved (Figure 8).

In order to confirm the histopathological diagnosis due to the rarity of the case and the age of the patient, an additional consultation from a second pathologist was requested. The second pathologist examined the same tissue specimen, and the investigation revealed several abnormal microcystic or tubular spaces, lined by immature glandular columnar epithelium, sometimes also present with goblet cells. A few islands of immature squamous cell epithelium were also recognized. Areas of mature cartilage with smooth outlines were observed, resembling the cartilaginous petals of the bronchi. Depending on the location, the growth of neuroepithelial-type cells was noticed, either completely immature cells in a compact arrangement or differentiated cells arranged into true rosettes, some of which had a true lumen.

In one location, immature epithelioid cells (probably neuroepithelial) grew sparsely in aggregates within a loose fibrous layer. These cells were negative in PLAP, AFP, and CD30, with only focal positivity for keratin 7 (CK 7).

Extensive ulceration with an inflammatory overlay was observed on the surface, as well as quite dense inflammatory infiltrates with lymphocytes, plasma cells, and polymorphonuclears. Immunohistochemically, almost all epithelial elements showed positivity for keratins AE1/AE3 (CK AE1/AE3), and many of the epithelia showed keratin 7 (CK 7) positivity. Positivity for P40 was observed in the slightly immature squamous epithelium, while the same positivity indicated the presence of basal-myoepithelial cells in cystic-tubular slit-like spaces with immature pseudostratified columnar epithelium.

In the same locations, the columnar epithelium showed nuclear positivity for TTF1. Many immature epithelia showed CD99 positivity, as well as mature cartilage. Also, focal positivity was observed for CD99 in the neuroepithelia, mainly in those with a rosette-like layout compared to those in a compact layout.

In the neuroepithelia, focal positivity was observed for synaptophysin and weaker for NSE, while SOX10 and GFAP positivity was rare. Maturing cartilage and a few epithelial and neuroepithelial cells showed S100 positivity.

The abovementioned immunohistochemical findings suggested the extremely rare presence of an immature malignant teratoma.

Furthermore, the patient was referred to pediatric oncologists for further examination in order to investigate the possibility of a primary occult malignancy. The brain MRI and the full body PET/CT scan did not reveal any areas of higher metabolic activity, either at the tumor site or elsewhere, thus not supporting the theory of metastatic teratoma, at least for the time being.

The molecular examination (Comprehensive Leading Array for Identification of Fusion Yield, C.L.AR.I.F.Y.) of the initial tissue specimen showed compatibility with Ewing sarcoma.

**Figure 9:**
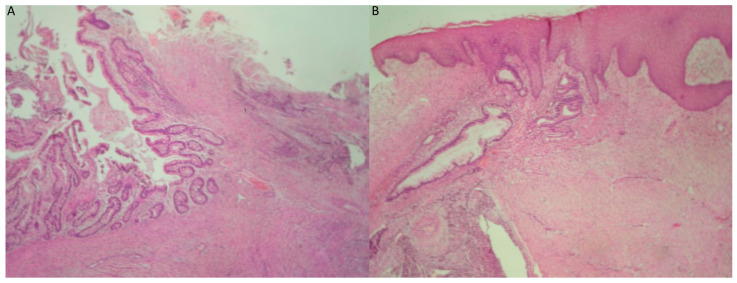
(**A**) Immature teratoma of the gingiva. Mature colonic mucosa is observed (left), in close proximity to immature neuroepithelial elements (hematoxylin–eosin ×40). (**B**) Mature gastric, pyloric-type tissue is observed under the stratified squamous epithelium of the gingiva (hematoxylin–eosin ×40).

**Figure 10:**
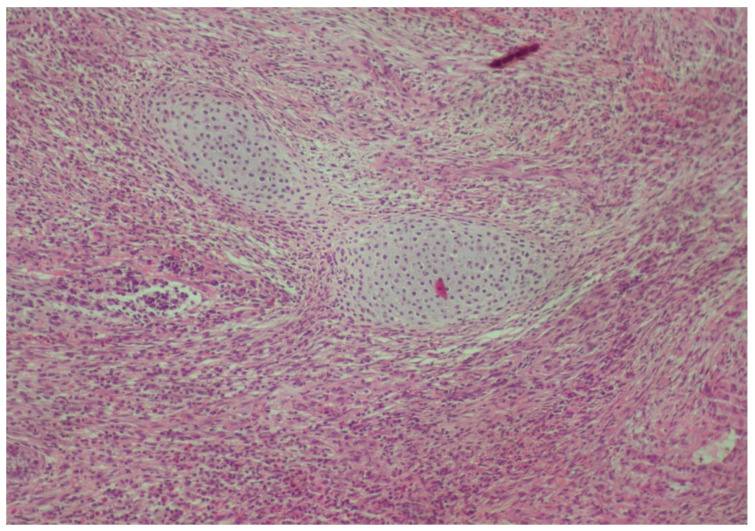
Immature cartilage in the center is admixed with neuroepithelial tissue (hematoxylin–eosin ×100).

Six months after the initial excisional biopsy, the lesion reappeared. In the relapsed lesion, a new incisional biopsy was performed and the histopathological examination revealed immature malignant teratoma (Figure 9 and Figure 10).

The patient was immediately referred for a CT scan, which showed a round mass (dimensions: mesiodistal 1.2 cm and buccolingual 0.8 cm).

**Figure 11:**
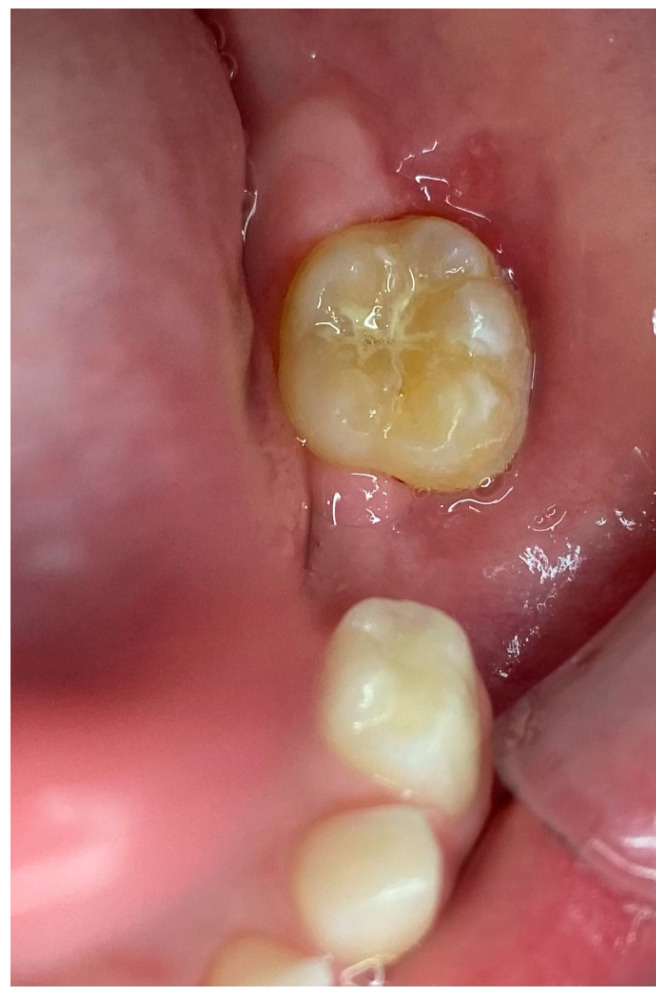
Postoperative clinical imaging. Tooth #75 was extracted, and tooth #36 had fully erupted.

The pediatric oncologists suggested that the patient receive chemotherapy to eliminate the possibility of an unidentified primary site of the metastatic malignant teratoma. Following chemotherapy, the rest of the teratoma, along with the adjacent tooth #75 (Figure 11), was removed, and the histopathological examination revealed a mature teratoma.

The patient remains disease-free, for the time being, under constant monitoring, with immature malignant teratoma of the gingiva being the primary diagnosis. At the time this article was written, the patient had been under monitoring for one year.

## 3. Discussion

We describe a rare case of intraoral malignant teratoma, and an extensive literature review revealed that our case is the first ever reported.

Ewing’s sarcoma (ES) is a relatively common malignancy in minors that involves the bones and less frequently affects extraosseous sites [5,6,7]. Ewing’s sarcoma accounts for 2% of all childhood malignancies [8] and up to 15% of all malignant bone diseases [9]. It constitutes an aggressive malignancy, with an estimated 15% to 28% of cases presenting with metastases at diagnosis [8,10]. However, when occult micrometastases are taken into account, this percentage increases to 80% [11]. The tumor has a male-to-female prevalence ratio of 2.1–2.4:1 [7,11], and is ten times more common in white children than in black children [12]. Furthermore, ES belongs to the broader classification of small round blue cell tumors [12].

Adamantinoma-like Ewing sarcoma (ALES) constitutes a rare variant of Ewing sarcoma. ALES primarily affects the head and neck region (74% of the confirmed cases) [13]. In this specific localization, ALES may be confused with other small round blue cell tumors and basaloid carcinomas [13]. The immunohistochemical profile of ALES includes positive membranous CD99 staining as well as positivity for cytokeratin, p63, and p40. The diffuse cytokeratin expression and positivity for high-molecular-weight cytokeratins is considered pathognomonic [13]. Diffuse positivity for p63 or p40 is not frequently observed in classical Ewing sarcoma [14,15]. Furthermore, ALES may exhibit positivity for neuroendocrine markers (such as synaptophysin); however, these markers are usually expressed in salivary localizations [16]. ALES is negative for S100, SMA, desmin, WT1, and NUT1 [13].

Synovial sarcoma (SS) is another relatively common sarcoma affecting the soft tissues [17,18]. This lesion accounts for 5% to 10% of soft tissue sarcomas and is most common in the extremities, especially the lower extremities [19]. Synovial sarcoma of the head and neck area is rare (6–7% of all cases) [17,18,20]. The neck, hypopharynx, and parapharyngeal space are most commonly affected [21]. In contrast, the oral cavity is rarely affected [18]. SS affects mostly minors, with a mild male predilection [18,22]. SS does not arise from true synovial cells but rather from multipotent mesenchymal cells [17]. Lesions in the head and neck have a worse prognosis than those in other sites [22]. SS is divided into four categories, with biphasic being the most frequently observed [21]. The immunohistochemical examination is crucial for differential diagnosis; SS is positive for epithelial markers, such as cytokeratin, epithelial membrane antigen (EMA), as well as vimentin, and is usually negative for CD34 and FLI-1 [23].

Teratomas are a subtype of germ cell tumors and their emergence outside the common gonadal and midline locations is exceptional [24]. A very rare case was reported of a recurrent immature ovarian teratoma that progressed into primitive neuroectodermal tumor/extraskeletal Ewing sarcoma (immunohistochemical positivity for CD99 and CD56) [4].

Another case, similar to ours, reports a single soft-tissue mass in the thigh of a 27-year-old man, with areas of both mature and immature teratoma, without other clinical or radiological evidence of involvement, apart from lymph node involvement [25].

The literature supports the notion of mature teratomas turning directly into malignancies [4,26,27]. The hypothesis could be elaborated that mature teratomas may include areas of immature teratoma or that an overlap could exist, where mature teratoma develops into sarcoma while an adjacent immature teratoma is present.

Of course, the case could be made that the tumor was metastatic, induced by cancer stem cells, manifesting epithelial-to-mesenchymal transition [28] In this clinical scenario, any radiographical examination would be rendered useless at such an early stage of the disease, as the presence of a small number of cancer stem cells and cancer cells in the primary localization would not be detected.

## 4. Conclusions

This case illustrates the necessity for regular follow-ups and the crucial role of the dentist, and in this case of the pediatric dentist, to promptly diagnose the underlying disease. Furthermore, genetic screening may assist in detecting high-risk populations, even from a young age. Especially in such complex histopathological cases, the importance of cooperating with experienced oral and maxillofacial pathologists is highlighted, as well as the necessity of subjecting the tissue specimen to examination by different, independent pathologists to minimize the margin of error. Both immature malignant teratoma and metastatic teratoma constitute very rare clinical entities, especially when localized in the oral cavity, and the patient remains under close monitoring to detect any relapse.

## Data Availability

The original data presented in the study are included in the article, further inquiries can be directed to the corresponding author.
